# Water-soluble pyridinium redox mediators for pH-swing CO_2_ capture

**DOI:** 10.1039/d5sc04731e

**Published:** 2025-09-24

**Authors:** Eloi Grignon, Zhangfei Su, Jiang Tian Liu, Armanda Lima, Andrew Wang, Parisa Karimi, Shuai Chen, Dwight S. Seferos

**Affiliations:** a Department of Chemistry, University of Toronto, Lash Miller Chemical Laboratories 80 St. George Street Toronto Ontario M5S 3H6 Canada; b Department of Chemical Engineering and Applied Chemistry, University of Toronto 200 College Street Toronto Ontario M5S 3E5 Canada dwight.seferos@utoronto.ca; c Clean Energy Innovation Research Centre (CEI), National Research Council Canada 2620 Speakman Drive Mississauga Ontario L5K 2L1 Canada shuai.chen@nrc-cnrc.gc.ca

## Abstract

Electrochemical pH-swing processes offer a promising route for energy-efficient CO_2_ capture but require robust redox mediators. This paper reports three water-soluble 4-substituted pyridinium redox mediators (BzM, BzSP, AcSP) for electrochemical pH-swing carbon capture and systematically evaluates their performance through H-cell and flow cell configurations. Highly water-soluble mediators bearing a propylsulfonate group can be readily synthesized in one step on a 20 g scale. We find that the inclusion of a benzoyl group at the 4-position is critical for attaining high current densities. The zwitterionic BzSP exhibits around 90% CO_2_ capture efficiency and optimal CO_2_ capture capacity (102 kJ mol_CO_2__^−1^) in a flow cell. These findings establish molecular design principles for pyridinium-based mediators in energy-efficient carbon management.

## Abstract 2

Water-soluble pyridinium redox mediators for pH-swing CO_2_ capture demonstrate 102 kJ mol_CO_2__^−1^ capture capacity in a flow cell, establishing molecular design principles for pyridinium-based mediators in carbon management.

## Graphical abstract

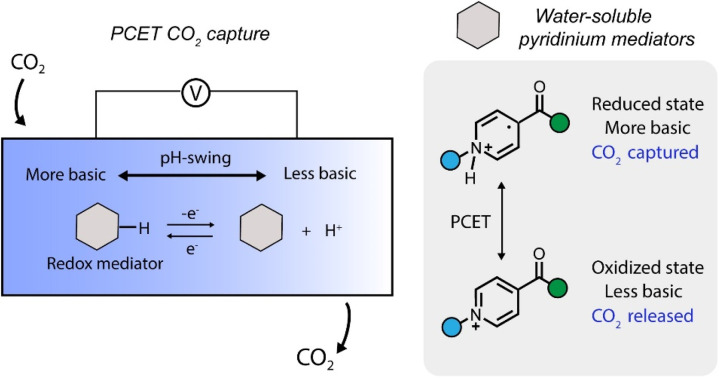

## Introduction

Carbon capture is an attractive strategy for mitigating anthropogenic CO_2_ emissions to curb global climate change. Developing this technology is especially important for sectors where emissions are hard to abate through other means, such as the cement or steel industry. Currently, most deployed carbon capture systems rely on liquid sorbents that must be thermally regenerated when saturated.^1^ This energy-consuming step substantially raises operational costs, thus presenting a barrier to the widespread adoption of the technology. In addition, the regeneration step has a large CO_2_ footprint, thus partially offsetting the benefits of the capture process.^2^ As such, developing more sustainable approaches to carbon capture is a timely challenge.

Recently, carbon capture methods relying on electrochemical regeneration have garnered significant attention as sustainable, efficient, and safe alternatives to the traditional thermal approach.^1–3^ In many of these methods, low-cost electricity is used to reversibly interconvert a molecular redox mediator between two redox states, where one state leads to CO_2_ capture while the other leads to CO_2_ release.

**Fig. 1:**
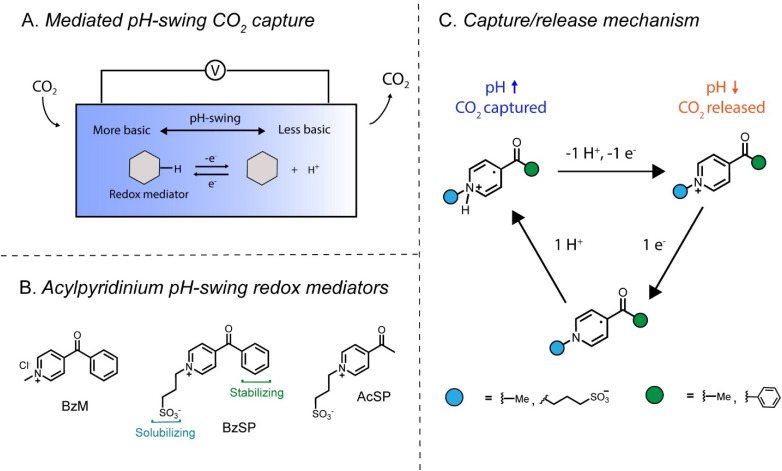
(A) General scheme for redox-mediated pH-swing carbon capture in one compartment. (B) Acylpyridinium redox mediators used in this work. (C) Mechanism of CO_2_ capture with pyridinium mediators.

In the simplest redox-mediated electrochemical approach, direct binding, an initially unreactive mediator is reduced to a more nucleophilic state where it can temporarily bind electrophilic CO_2_.^4^ Upon reoxidation of the mediator, its nucleophilicity is lowered and CO_2_ is released. This strategy has been demonstrated in non-aqueous media with disulfides,^5^ pyridines,^6^ and quinones.^7^ However, in this method, the use of organic solvent as the electrolyte is undesirable from a cost and safety standpoint. A more elaborate approach, pH-swing, involves the reversible switching of the mediator's basicity through proton-coupled electron transfer (PCET).^8^ The principle of pH-swing CO_2_ capture/release is illustrated in Fig. 1A. The reduction of the redox mediator consumes protons in the electrolyte, causing an increase in the electrolyte pH and the capture of CO_2_ as carbonate or bicarbonate. Upon reoxidation, the mediator releases protons, leading to a decrease in the electrolyte pH and the release of CO_2_. A key advantage of the pH-swing strategy is that the capture/release process occurs in water, which reduces operational costs, increases the system's safety, and enables high current densities. Several pH-swing mediator motifs have been reported, such as quinones,^9,10^ phenazines,^11–15^ 1-aminopyridinium,^16^ riboflavin,^17^ and guanidines.^18^ However, the field is still nascent, and the exploration of further molecules is critical to enlarging the library of viable mediators.

Herein, we report pH-swing carbon capture based on 4-substituted acylpyridinium mediators. These molecules are synthesized in 1–2 steps and are isolated through a simple filtration, which is ideal for scale-up. The acylpyridinium molecular family was previously investigated for aqueous redox flow batteries by Sevov *et al.* and found to induce pH changes due to proton abstraction.^19^ In this work, we exploit this property to mediate cyclical CO_2_ capture.

The pyridinium mediators are synthesized in their oxidized form and can be reduced through PCET to form stable radicals. The solubility and radical stability of the mediators can be influenced by the group at the *N*- and 4-positions, respectively. In this study, we synthesized and studied three mediators: 4-benzoyl-1-methylpyridin-1-ium chloride (BzM), 3-(4-benzoylpyridin-1-ium-1-yl)propane-1-sulfonate (BzSP), and 3-(4-acetylpyridin-1-ium-1-yl)propane-1-sulfonate (AcSP) (Fig. 1B). The proton-coupled redox behavior of the mediators and their CO_2_ capture/release mechanism is illustrated in Fig. 1C. During reduction, a pyridinium radical is formed, which takes up protons from the solution and enables CO_2_ capture. In the subsequent reoxidation stage, the pyridinium radical undergoes electron loss and deprotonation, triggering CO_2_ release. Overall, the mediators lower the energetic input required to generate a pH-swing, thus enabling energy-efficient CO_2_ capture.

## Results

### CVs of pyridinium redox mediators

**Fig. 2:**
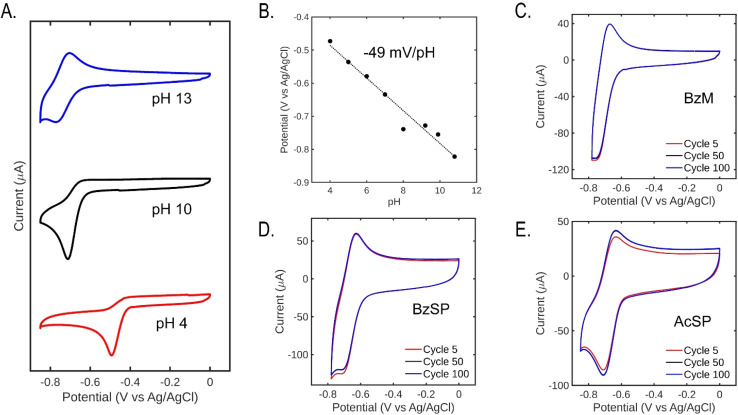
Cyclic voltammetry of 5 mM pyridinium mediators at 100 mV s^−1^ under argon. (A) Cyclic voltammograms of BzM in various buffers with 1 M KNO_3_. (B) Pourbaix diagram of BzM. (C–E) Long-term cyclic voltammograms in unbuffered 1 M KNO_3_ of BzM, BzSP, and AcSP.

Cyclic voltammetry (CV) in 1 M KNO_3_ (aq.) was used to screen the pH-swing mediators' electrochemical properties before capture/release tests. We started our exploration of this family of molecules with the previously reported BzM (Fig. 1B).^19,20^ A conservative potential window was employed to limit the mediators to one reduction, thus avoiding the known reversibility issues associated with further reduced acylpyridinium products (Fig. S8).^19^ The redox process of BzM was first studied in solutions of varying pH to understand the influence of pH on reversibility and redox potential. The redox process is reversible at pH values above 11 but not at pH values below 10 (Fig. 2A), which implies a chemical step (protonation) when the concentration of H^+^ is sufficiently high (*i.e.* when pH < p*K*_a_). Moreover, CVs over a wide pH range show that the potential of BzM's first reduction is pH-dependent, with a change of approximately −49 mV pH^−1^, confirming a 1 e^−^/1 H^+^ proton-coupled electron transfer (Fig. 2B).^21^ This is consistent with a previous study of BzM.^20^ Taken together, these findings confirm that BzM can be electrochemically transformed into a base, which is the fundamental requirement of a pH-swing mediator.

A high mediator solubility in all redox stages is important to maximize the capture solution's CO_2_ capacity. BzM's solubility comes primarily from its positive charge, which disappears during the reduction step (unless protonated). Thus, to avoid possible solubility issues with BzM, we also synthesized BzSP, which bears a permanently anionic propylsulfonate group (Fig. 1B). These substituents have been used extensively as solubilizing groups for bispyridiniums (*i.e.* viologens) in aqueous redox-flow batteries.^22–26^ Finally, AcSP was synthesized as a control mediator to evaluate the influence of the substituent at the 4-position (Fig. 1B). All mediators showed the expected signals in their NMR (Fig. S1–S6) and FTIR (Fig. S7) spectra. In unbuffered 1 M KNO_3_ (aq.), a clear redox event is observed at −0.71 V *vs.* Ag/AgCl for BzM and −0.69 V for BzSP and AcSP (Fig. 2C–E). The reduction of pyridinium mediators occurs within the pyridinium ring, and this structural feature is consistent in the three molecules, hence, the reduction potentials are similar. Prolonged CV experiments show slight variations in current in the early cycles, which is likely a result of local pH changes. However, the redox process quickly stabilizes and remains unchanged for at least 100 cycles. Finally, the reduction potentials of BzSP and AcSP also exhibit a strong pH-dependence, confirming the presence of PCET in these mediators (Fig. S9).

### Carbon capture/release experiments in H-cell

Carbon capture/release experiments were carried out in an H-cell system (Fig. S10). The working electrode (WE) compartment contained 0.1 M pyridinium mediator and 1 M KNO_3_, while the counter electrode (CE) compartment contained 0.1 M K_3_Fe(CN)_6_ and 0.2 M K_4_Fe(CN)_6_, which served as an auxiliary redox couple. Pt coils were employed as WE and CE, with a Ag/AgCl (sat. KCl) as the reference electron (RE), which was located in the WE compartment. A pH sensor and CO_2_ sensor provided real-time data on the capture/release process. Throughout the experiment, the solution in the WE compartment was continuously purged with a CO_2_ : N_2_ mixture (10 : 90, v/v), which simulates an industrial flue gas stream. A CO_2_ concentration in the gas outlet lower than 10% corresponds to CO_2_ capture, while CO_2_ concentrations in the gas outlet higher than 10% indicate CO_2_ release. In the test of BzSP and AcSP, the WE and CE compartments were separated by a Nafion 117 cation exchange membrane (CEM), which allows cation transport between the two compartments during the test. However, since BzM is a cation, prolonged experiments result in its migration across the CEM to the CE compartment. This crossover phenomenon causes progressive depletion of BzM in the working electrolyte, ultimately diminishing the CO_2_ capture capacity. Hence, in the test of BzM, a Fumasep FAA-3-PK-75 anion exchange membrane (AEM) was used to assemble the H-cell, and the electrolyte in the CE compartment comprised 0.2 M FeCl_2_ and 0.1 M FeCl_3_.

Fig. S11 plots the CVs of three pyridinium mediators measured in the H-cell. The reduction current of BzM and BzSP is larger than AcSP, which follows the same trend as the CVs measured in diluted solution using the GC electrode, indicating that stabilizing benzoyl moiety facilitates electron transfer kinetics by increasing the stability of the reduction product. The reduction potential of BzSP (−0.78 V) is more positive compared to AcSP (−0.82 V) and BzM (−0.85 V with Nafion membrane), which is also in the same trend as the results in a diluted solution.

**Fig. 3:**
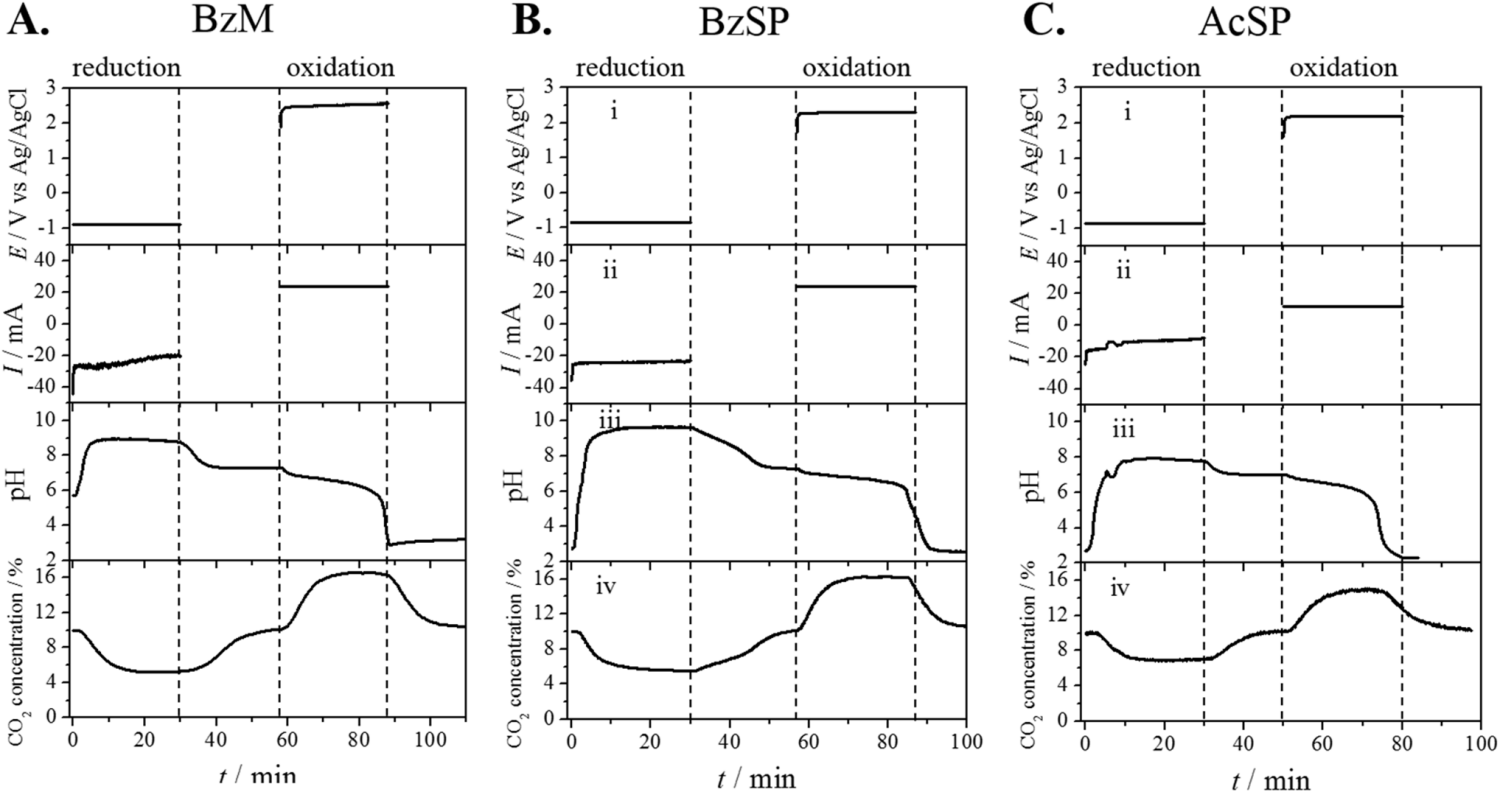
H-cell CO_2_ capture/release tests. (A–C) CO_2_ capture/release results in the H-cell, (A) BzM, (B) BzSP, (C) AcSP. Fumasep AEM was used for BzM, and Nafion CEM was employed in the test of BzSP and AcSP. The pyridinium mediators were reduced using a constant potential and were reoxidized using a constant current. (i) Applied potential on the Pt WE, (ii) current of the Pt WE, (iii) pH of electrolyte in the WE compartment and (iv) CO_2_ concentration in the gas outlet.

Fig. 3A plots the results of the CO_2_ capture/release test using BzM as the redox mediator in the H-cell. During the experiment, the mediator was first reduced to generate a base, thus taking up protons from the solution and raising the pH. During this stage, acidic CO_2_ was captured by the solution and stored as (bi)carbonate ions. Based on the CV (red curve in Fig. S11A) measured in the H-cell, the reduction potential for BzM was set at −0.90 V *vs.* Ag/AgCl. This potential was strategically set below the reduction potential of BzM, and above the hydrogen evolution reaction (HER) potential on the Pt surface, thereby suppressing competitive H_2_ generation on the Pt coil. In Fig. 3A, the BzM reduction current was approximately −24.0 mA, accompanied by a gradual pH increase in the electrolyte. After 30 minutes of reduction, the potential was held at open circuit for 30 minutes to facilitate complete CO_2_ capture. The solution pH decreased to 7.3 during this additional invasion period.

BzM was subsequently reoxidized under constant-current conditions at 24.0 mA, matching the average BzM reduction current. This condition ensured that the total charge passed through the Pt WE during the reduction and oxidation stages was balanced. The potential during BzM reoxidation was around +2.5 V *vs.* Ag/AgCl, demonstrating that the reoxidation process is energetically demanding and requires external energy input. Concurrent with reoxidation, the pH within the WE compartment underwent a pronounced decrease, falling from 7.3 to 2.9. This acidification of the electrolyte enabled the efficient release of captured CO_2_ from the solution. However, after the test, it was found that the OH^−^ anions generated in the reduction stage migrated across the AEM and formed Fe(OH)_2_ and Fe(OH)_3_ precipitates in the CE compartment. Hence, BzM is not a good candidate for the pH-swing CO_2_ capture/release.

Fig. 3B and C show the results of the CO_2_ capture/release test using BzSP and AcSP as the redox mediators. Based on the CVs (Fig. S11B & C) measured in the H-cell, the reduction potentials for BzSP and AcSP were set at −0.85 and −0.90 V *vs.* Ag/AgCl, respectively. The average BzSP reduction current was −23.9 mA, higher than AcSP's −12.0 mA. Following 30 minutes of reduction, the potential was held at open circuit for 30 minutes to ensure complete CO_2_ capture and proton regeneration. The mediators were then reoxidized in constant-current mode using currents equal to the absolute values of their respective reduction currents. BzSP demonstrates a better CO_2_ capture capacity compared to AcSP, as evidenced by three key metrics: (1) a larger current in the reduction stage, (2) a more alkaline post-reduction solution (pH 9.7 for BzSP *vs.* 7.9 for AcSP), and (3) greater CO_2_ concentration change in the gas outlet. These collective findings, as systematically demonstrated in Fig. 3B and C, establish BzSP's higher capacity in the pH-swing CO_2_ capture system. The amplified pH fluctuation and corresponding gas concentration changes directly correlate with its improved charge transfer efficiency.

To distinguish whether CO_2_ capture originates from the reduction of the pyridinium mediators or competing HER, a control experiment was performed in the H-cell containing exclusively 1 M KNO_3_ in the WE compartment. Fig. S12A presents the carbon capture result obtained at a reduction potential of −0.85 V, which matches the potential applied for BzSP. The reduction current of 1 M KNO_3_ without pyridinium mediator is between 1 and 2 mA, significantly lower than that observed in the electrolyte with 0.1 M BzSP, indicating that HER is quite slow at this potential. Correspondingly, the change in pH and CO_2_ concentration is also small in the solution without the pyridinium mediator (Fig. S12B & C). The results in Fig. S12 indicate that the faradaic current in Fig. 3 predominantly arises from the reduction of the pyridinium mediators, with concomitant pH increase and CO_2_ capture being directly associated with this redox process rather than HER.

### Carbon capture/release experiments in a flow cell

**Fig. 4:**
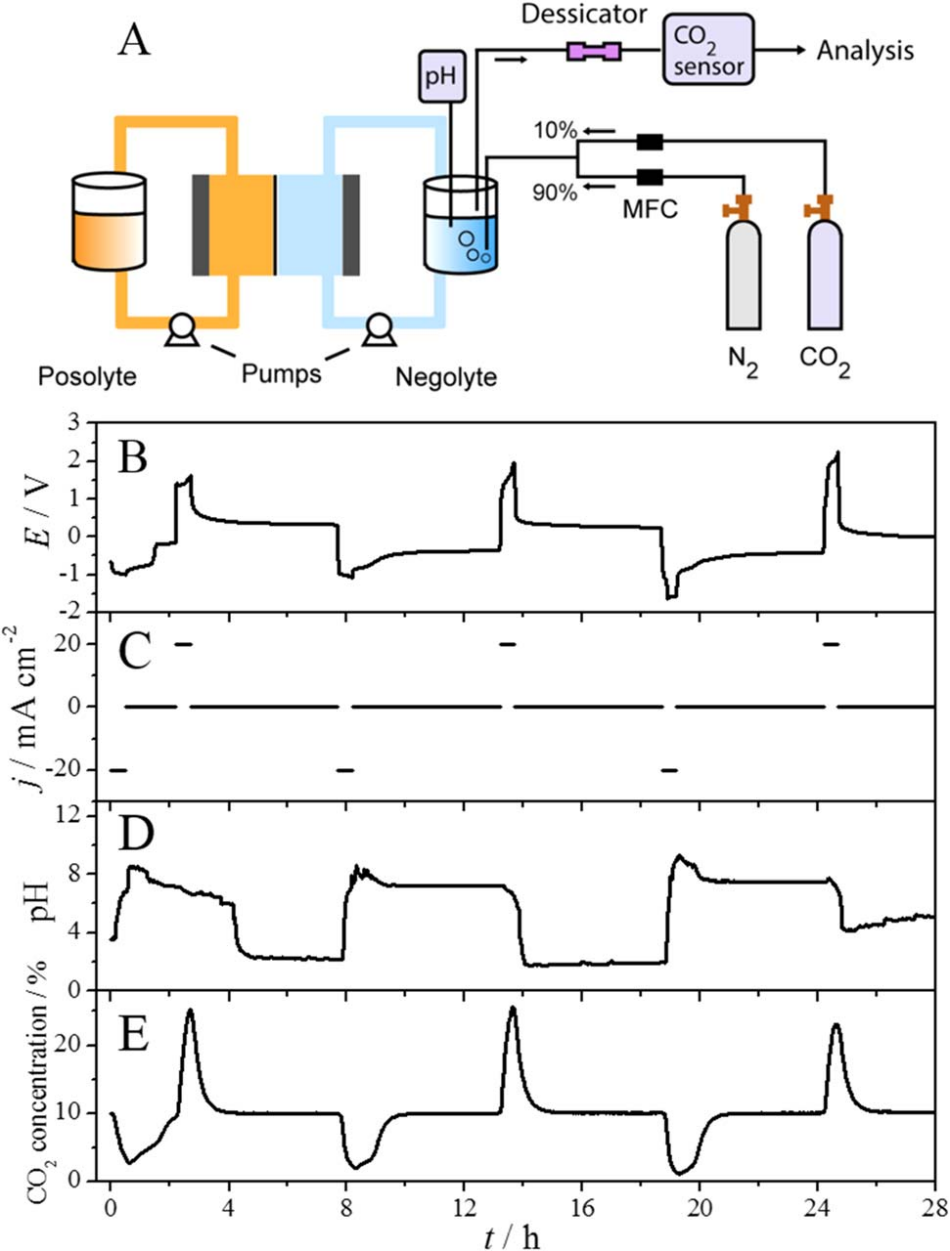
(A) Schematic diagram of the flow cell system used in the pH-swing CO_2_ capture/release test. (B–E) Variation of the cell potential (B), current density of WE (C), pH of negolyte (D) and CO_2_ concentration in the gas outlet (E) as a function of time during the CO_2_ capture/release test.

From Fig. 3, it can be concluded that BzSP shows the best performance in the CO_2_ capture/release test. As the next step, we scaled up the synthesis of this champion mediator to a 20 g scale (see SI) and tested its energy efficiency for the CO_2_ capture/release in a more commercially relevant flow cell. The schematic diagram of the flow cell setup is displayed in Fig. 4A. The cell was assembled with a Nafion 117 membrane and 4 cm^2^ carbon papers (Sigracet 22AA) as the working and counter electrodes. The negolyte contained 25 mL of 0.1 M pyridinium mediator in 1 M KNO_3_, and the posolyte contained 40 mL of 0.1 M K_3_Fe(CN)_6_ and 0.2 M K_4_Fe(CN)_6_. The negolyte was continuously purged with a CO_2_ : N_2_ mixture (10 : 90, v/v), and the pH of the negolyte was monitored by a pH sensor. Two peristaltic pumps were used to circulate the negolyte and posolyte into the cell during the test.

Fig. 4B–E plots the results of pH-swing CO_2_ capture/release in the flow cell configuration. The redox cycling protocol applied reduction and oxidation current of ±20 mA cm^−2^, respectively (Fig. 4C), with both stages maintained for 30 minutes to ensure equivalent total charge between the redox processes. In the first and second cycles, during the reduction stage, the cell potential was around −1.0 V (Fig. 4B), and the pH of the negolyte rose from 3.6 to 8.5 (Fig. 4D) concomitant with CO_2_ concentration dropping from 10% to 2.9% (Fig. 4E), demonstrating CO_2_ capture through proton consumption. A subsequent equilibration period at OCP allowed for further CO_2_ uptake, as shown by a gradual decrease in pH to 7.2. In the reoxidation stage, the cell potential was between 1.4 and 1.6 V, triggering a decrease in pH to 2.3 and an increase in CO_2_ concentration to 25.2%, thereby confirming CO_2_ release through proton regeneration.

In the CO_2_ concentration plot, the CO_2_ release peak is narrower than the capture peak, and the maximum CO_2_ concentration change in the oxidation stage is larger than that observed in the reduction stage. These results indicate that the rate of CO_2_ release is faster than the rate of CO_2_ capture. This difference arises because CO_2_ capture is controlled by CO_2_ absorption kinetics, while CO_2_ release is constrained by the applied current density.^13^

At the end of the second cycle, the reoxidation potential exhibits a sudden increase from 1.7 V to 1.9 V. This increase suggests that BzSP may be depleted within the electrolyte. Subsequently, during the third cycle, the reduction potential decreases to −1.6 V while the oxidation potential increases further to 1.9 V. The elevated cell potential indicates that water splitting, which requires higher energy than BzSP reduction, becomes the dominant reaction. The depletion of BzSP during cycling is further supported by post-mortem CV analysis from H-cell capture/release tests, where the redox peaks of BzSP greatly diminish after three cycles (Fig. S13). These results demonstrate that BzSP remains stable for approximately 16 hours, corresponding to two full cycles. The pH of the negolyte at the end of the third cycle is higher than that measured at the end of the first and second cycles. This increase results from BzSP degradation reactions, which consume H^+^ ions (see the next section for mechanistic studies of BzSP reduction).

The theoretical CO_2_ capture capacity of the BzSP solution can be calculated as:

1 *C* = *nc*_BzSP_*V*

where *n*, *c*_BzSP_, *V* are the number of electrons transferred per BzSP molecule during reduction/oxidation (*n* = 1), concentration of BzSP, and volume of negolyte, respectively. Following 30 minutes of reduction at 20 mA cm^−2^, approximately 60% of the theoretical BzSP capacity was reduced. The amount of CO_2_ captured/released by BzSP can be calculated using the area of peaks in the CO_2_ concentration plot (Fig. 4E). The CO_2_ capture/release efficiency (CE) can be determined as the ratio between the moles of CO_2_ captured/released and moles of electrons transferred as:2
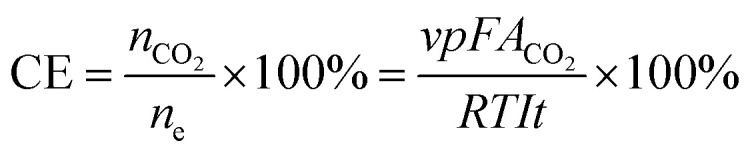
where *v*, *p*, *F*, *A*_CO_2__, *R*, *T*, *I*, *t* are the flow rate of effluent gas, pressure, Faraday constant, area of the CO_2_ peak, ideal gas constant, temperature, current, and time, respectively. The energy efficiency (EE) of the flow cell can also be calculated as:3
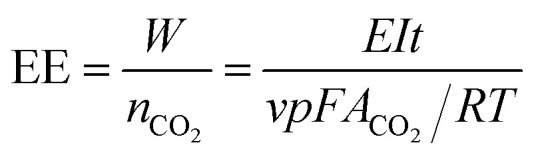


The CO_2_ capture/release ratio (*C*/*R*) in one reduction–oxidation cycle is determined as:4
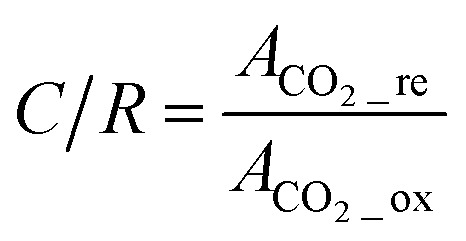
where *A*_CO_2__re_ and *A*_CO_2__ox_ are the area of the CO_2_ peak in the reduction and oxidation steps, respectively.

During the first two cycles, the CO_2_ capture/release efficiency is approximately 90% in the reduction stage and surpasses 100% in the oxidation stage (Table 1). The high efficiency in the oxidation stage may result from faster kinetics during CO_2_ release. In the third cycle, where water splitting occurs, the efficiency of the reduction and oxidation stages becomes similar. In the reduction stage of the first two cycles, the energy efficiency of CO_2_ capture is 102 kJ mol_CO_2__^−1^, and in the oxidation stage, the energy efficiency of CO_2_ release is 136 kJ mol_CO_2__^−1^. For the third cycle, the energy input increases for both stages due to the higher energy demand of water splitting compared to BzSP reduction and reoxidation.

**Table 1:** CO_2_ capture/release efficiency, energy efficiency and CO_2_ capture/release ratio using 0.1 M BzSP in the flow cell at a current density of 20 mA cm^−2^

	CO_2_ capture/release efficiency/%	Energy efficiency/kJ mol_CO_2__^−1^	CO_2_ capture/release ratio/%
Reduction cycle 1	90	102	88
Oxidation cycle 1	102	136	
Reduction cycle 2	89	108	84
Oxidation cycle 2	107	136	
Reduction cycle 3	94	143	98
Oxidation cycle 3	95	176	

The performance and energy efficiency of BzSP is compared with other reported redox mediators in Table S2. The reduction potential and CO_2_ capture energy efficiency of BzSP are competitive with state-of-the-art redox mediators.^9,11,13,16^ However, a significant energy cost is incurred during the oxidation stage, resulting in an overall energy efficiency that is currently higher than literature values for other mediators. To enhance the viability of our system, we are actively optimizing the oxidative process to improve its energy efficiency.

### Mechanistic studies

**Fig. 5:**
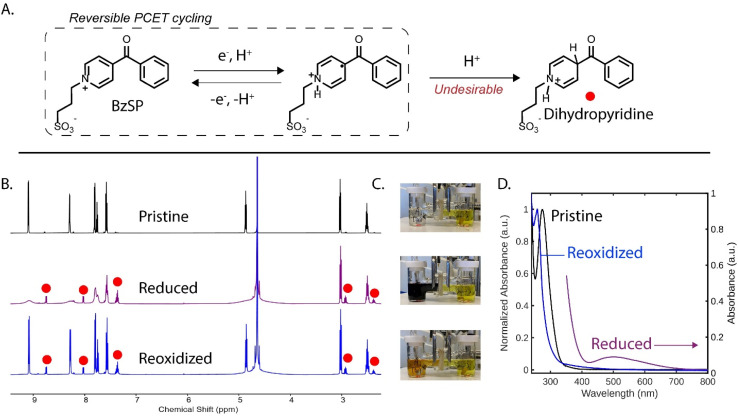
(A) Mechanism for BzSP-mediated CO_2_ capture, including degradation reaction to dihydropyridine. (B) ^1^H NMR spectra (D_2_O, 500 MHz) of 0.1 M BzSP in 1 M KNO_3_ at different stages of the cycling process. (C) Photographs of the BzSP solution (left compartment) at each stage. (D) UV-visible absorption spectra at each stage. The pristine and reoxidized spectra are normalized, while the reduced spectrum is not. A large absorbance below 400 nm in the reduced spectrum is due to the high concentration of active species, which is necessary to observe the radical band.

To gain insight into the mechanism of BzSP, we carried out a similar CO_2_ capture/release experiment using D_2_O as the electrolyte solvent, thus enabling ^1^H NMR analysis of the mediator at each stage of the process (Fig. 5B). Initially, the BzSP mediator is in its pristine state and its NMR peaks are aligned with those found in pure D_2_O. Following reduction, a broadening of the aromatic peaks is observed, consistent with the formation of radicals. This is further supported by UV-vis spectroscopy of the deep purple reduced capture solution (Fig. 5C), which shows a broad band centered at 500 nm (Fig. 5D).^27^ Exposure of this solution to CO_2_ leads to a new peak at 160 ppm in the ^13^C NMR spectrum, which is attributed to HCO_3_^−^, thus confirming successful sequestration of CO_2_ (Fig. S14).

The NMR analysis is particularly useful for monitoring degradation. During reduction, small side peaks emerge in both the aromatic and aliphatic regions, indicating the formation of a new product with a very similar structure to BzSP (Fig. 5B). These peaks are still observed following oxidation and therefore represent a small loss of mediator over one capture/release cycle (approximately 11%). From mass spectrometry (Fig. S15) and further NMR studies (Fig. S16), the product is assigned as the dihydropyridine derivative of BzSP (Fig. 5A), which is consistent with previous work on pyridinium mediators.^19^ Interestingly, the dihydropyridine signal does not increase following the oxidation step, implying that the degradation primarily occurs in the early stage of reduction, where some of the mediator is in radical form and pH is relatively low. Thus, improving the radicals' stability to H^+^-induced degradation is crucial to improving the cycle life of this CO_2_ capture system. This has been achieved in organic solvents through careful design of the *N*- and 4-substituents.^28^

An experiment in D_2_O was also carried out for AcSP to understand its poor performance. Following reduction, the acetyl CH_3_ signal is absent from the solution's ^1^H NMR spectrum, indicating enolization and subsequent proton exchange with the solvent (Fig. S17), which is consistent with previous work.^19^ The ability of AcSP to enolize means that it is susceptible to aldol reactions, and therefore has more available degradation pathways than BzSP.

## Conclusions

Three water-soluble pyridinium-based redox mediators (BzM, BzSP, AcSP) have been synthesized and evaluated for electrochemical pH-swing CO_2_ capture/release, and we have identified structure–performance relationships, degradation mechanisms, and practical limitations. These molecules show reversible redox behavior and can electrochemically induce pH-swings, thus opening the door to CO_2_ capture. In H-cell tests, BzSP demonstrates optimal CO_2_ capture capacity, evidenced by higher reduction currents, larger pH swings, and greater CO_2_ concentration changes than AcSP and BzM. In a flow cell setup, BzSP exhibits stable operation for two full cycles (∼16 h), achieving ∼90% CO_2_ capture efficiency and ∼100% CO_2_ release efficiency. The energy requirement for CO_2_ capture is 102 kJ mol_CO_2__^−1^, however, CO_2_ release during oxidation requires significantly more energy (136 kJ mol_CO_2__^−1^), highlighting a key area for optimization. Degradation of the molecules through an acid-induced pathway was observed, which limits the mediator lifetime. Future work will aim to lower the energetic input of the oxidation stage, to improve the cycling life through rational molecular engineering, and to carry out experiments with more practically relevant gas streams, which include oxygen and impurities such as SO_*x*_ and NO_*x*_. Overall, this study establishes that the acylpyridinium family is suitable for electrochemically mediated CO_2_ capture and paves the way for the development of new pH-swing redox mediators.

## Author contributions

EG, DS, PK, and SC conceived the project. EG and JTL carried out the synthesis. ZS carried out FTIR while EG and JTL carried out all other chemical characterization. EG, JTL, ZS, SC, and AL carried out electrochemical studies. AW helped with system design. ZS and SC carried out long-term CO_2_ capture/release tests and analysis. EG and ZS drafted the manuscript and all authors contributed to revision. DS, PK, and SC supervised the project.

## Conflicts of interest

There are no conflicts to declare.

## Supplementary Material

SC-016-D5SC04731E-s001

## Data Availability

The authors confirm that the data supporting the findings of this study are available within the article and/or its SI. Supplementary information is available. See DOI: https://doi.org/10.1039/d5sc04731e.
