# Supplementary material for: Water-soluble pyridinium redox mediators for pH-swing CO2 capture
Source: Chem Sci. 2025 Sep 24;16(42):19702–10. doi: 10.1039/d5sc04731e (PMC12459621; doi:10.1039/d5sc04731e)
Supplement: SC-016-D5SC04731E-s001 [file SC-016-D5SC04731E-s001.pdf]

Supplementary Information (SI) for Chemical Science.
This journal is © The Royal Society of Chemistry 2025

Electronic Supplementary Information

Water-soluble Pyridinium Redox Mediators for pH-Swing CO_2 Capture

Eloi Grignon,[1 ‡] Zhangfei Su,[3 ‡] Jiang Tian Liu,[1] Armanda Lima,[3] Andrew Wang,[3] Parisa Karimi,[3] Shuai Chen,[3*] Dwight S. Seferos[1,2*]

[1]Department of Chemistry, University of Toronto, Lash Miller Chemical Laboratories, 80 St. George Street, Toronto, Ontario, M5S 3H6, Canada

[2]Department of Chemical Engineering and Applied Chemistry, University of Toronto, 200 College Street, Toronto, Ontario, M5S 3E5, Canada

[3]Clean Energy Innovation Research Centre (CEI), National Research Council Canada, 2620 Speakman Drive, Mississauga, Ontario, L5K 2L1, Canada

‡ These two authors contributed equally.

*Email address: shuai.chen@nrc-cnrc.gc.ca (S. Chen), dwight.seferos@utoronto.ca (D. Seferos)

Materials

4-Acetylpyridine (97%), 4-benzoylpyridine, iodomethane (99%), 1,3-propane sultone (98%), AmberLite™ HPR4100 anion exchange resin (Cl form), KNO_3 (≥99.0%), $K_3Fe(CN)_6$ (≥99.0%) and $K_4Fe(CN)_6$ (≥98.5%) were purchased from Sigma-Aldrich and used as received. Solvents used for chemical synthesis were purchased from Sigma-Aldrich and used as received. Ultrapure water (resistivity > 18.2 MΩ cm) from a Milli-Q water system was used to prepare aqueous solutions.

Methods

General instrumentation

NMR spectra were recorded at room temperature using a 400 MHz Bruker Advance III or a 600 MHz Agilent DD2 spectrometer and referenced to the solvent peak where possible. IR spectra were collected on an Agilent Cary 630 FTIR Spectrometer equipped with an ATR accessory, using 128 scans and an instrumental resolution of 16 cm^{-1}. Mass spectra were obtained on an Agilent 6538 UHD. UV-visible spectroscopy was carried out using an Agilent Cary 7000 UV-Vis-NIR Universal Measurement spectrophotometer. Electrochemical measurements were performed using a BioLogic SP-200 or a BioLogic VMP-3e multichannel potentiostat (BioLogic) connected to a current booster (VMP3B-20, 20A/20V).

Electrochemical characterization

Cyclic voltammetry was generally carried out in 1 M KNO_3 using a glassy carbon working electrode, an Ag/AgCl reference electrode, and a platinum mesh counter electrode under an Ar or CO_2 atmosphere. The concentration of pyridinium mediator was 5 mM. For the pH-dependent Pourbaix experiment, pre-made electrolytes consisting of phosphate buffer and 1 M NaCl were used, with the pH slightly adjusted using HCl or NaOH, as needed.

Carbon capture measurements in H-cell

CO_2 capture and release tests were carried out in an H-cell (Shanghai Chuxi Industrial Co. Ltd) under a 90:10 N_2:CO_2 atmosphere. The H-cell consisted of Pt coils for the working and counter electrodes, and Ag/AgCl for the reference electrode. In the test of BzSP and AcSP, a Nafion 117

cation exchange membrane was employed to separate WE and CE compartments; while for BzM, a Fumasep FAA-3-PK-75 anion exchange membrane was used. The WE compartment was filled with 0.1 M of the pyridinium mediators in 1 M KNO_3, while the CE compartment was filled with 0.1 M $K_3Fe(CN)_6$ & 0.2 M $K_4Fe(CN)_6$ for BzSP and AcSP, and 0.2 M $FeCl_2$ and 0.1 M $FeCl_3$ for BzM. The pH of the electrolyte in the WE side compartment was monitored using a LE 422 pH sensor (Mettler Toledo). The concentration of CO_2 in the mixed gas was monitored using a GMP251 CO_2 sensor (Vaisala).

Carbon capture measurements in a flow cell

The energy efficiency for the CO_2 capture/release cycles and stability of BzSP was measured using a flow cell. The flow cell (Dioxide Materials) is made of two Ti backplates with serpentine flow channels on both sides. Both cathode and anode were 4 cm^2 carbon papers (Sigracet 22AA), which were separated by a Nafion 117 membrane (Ion Power). The negolyte contained 25 mL of 0.1 M pyridinium mediator in 1 M KNO_3, and the posolyte contained 40 mL of 0.1 M $K_3Fe(CN)_6$ and 0.2 M $K_4Fe(CN)_6$. The negolyte was continuously purged with a CO_2:N_2 mixture (10:90, v/v). The flow rates of N_2 and CO_2 were 5.00 sccm and 0.555 sccm, respectively, which were controlled by a mass flow controller (Alicat Scientific). The pH of the negolyte was monitored by a pH sensor, and the concentration of CO_2 in the gas outlet was monitored using a GMP251 CO_2 sensor. Two peristaltic pumps (New Era Pump Systems, Inc.) were used to circulate the negolyte and posolyte into the flow cell during the test.

Synthetic Protocols

4-benzoyl-1-methylpyridin-1-ium chloride (BzM)

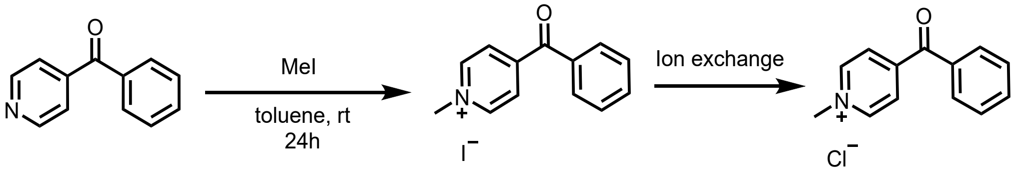

4-Benzoylpyridine (5 g, 27.3 mmol) was dissolved in toluene (20 mL) in a round-bottom flask. While stirring, iodomethane (2.55 mL, 41.0 mmol) was added to the flask. After 16 hours, the yellow precipitate was collected by vacuum filtration and washed with toluene, affording BzM as a yellow powder (2.88 g, 32 %).

To obtain the chloride form of BzM, the solid was redissolved in 3:1 H_2O/methanol and run through an ion exchange column (Amberlite), followed by evaporation of the solvent. Successful ion exchange can be confirmed by the absence of high-potential redox peaks in the CV of BzM.

^{1}H NMR (400 MHz, D_2O) δ 9.04 (d, J = 6.4 Hz, 2H), 8.32 (d, J = 6.2 Hz, 2H), 7.89 (d, J = 7.3 Hz ,2H), 7.83 (t, J = 7.5 Hz, 1H), 7.65 (t, J = 7.5 Hz, 2H), 4.52 (s, 3H). ^{13}C NMR (101 MHz, D_2O) δ 194.04, 151.25, 146.21, 135.35, 133.85, 130.58, 129.13, 127.53, 48.69. MS (ESI) m/z: calcd for $[C_{13}H_{12}NO]^{+}$: 198.09; found: 198.09.

3-(4-benzoylpyridin-1-ium-1-yl)propane-1-sulfonate (BzSP)

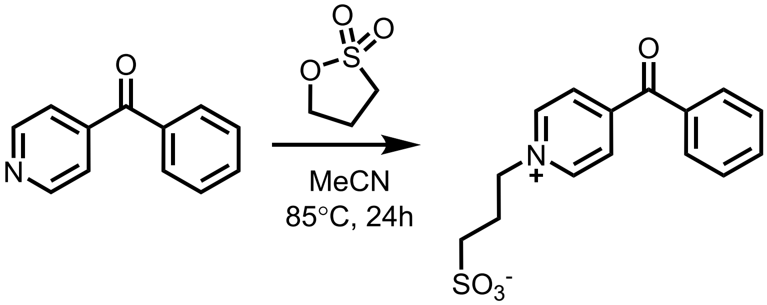

4-Benzoylpyridine (14.64 g, 80 mmol) was dissolved in acetonitrile (60 mL) in a round-bottom flask. While stirring, 1,3-propanesultone (9.76 g, 80 mmol) was added to the flask. The flask was fitted with a condenser, placed in an oil bath pre-heated to 85 °C, and left to stir at reflux. After 24 hours, the precipitate was collected by vacuum filtration, washed with acetonitrile, and dried under vacuum to afford BzSP as a white powder (23.74 g, 97 %).

^{1}H NMR (400 MHz, D_2O) δ 9.15 (d, J = 7.0 Hz, 2H), 8.37 (d, J = 6.6 Hz, 2H), 7.89 (d, J = 7.2 Hz, 2H), 7.83 (t, J = 7.5 Hz, 1H), 7.65 (t, J = 7.9 Hz, 2H), 4.92 (t, J = 7.5 Hz, 2H), 3.07 (t, J = 7.2 Hz, 2H), 2.56 (p, J = 7.2 Hz, 2H). ^{13}C NMR (101 MHz, D_2O) δ 193.96, 151.80, 145.56, 135.34, 133.84, 130.51, 129.09, 127.93, 60.34, 47.02, 26.23. MS (ESI) m/z: calcd for $[C_{15}H_{16}NO_4S]^+$: 306.08; found: 306.08.

<u>3-(4-acetylpyridin-1-ium-1-yl)propane-1-sulfonate (AcSP)</u>

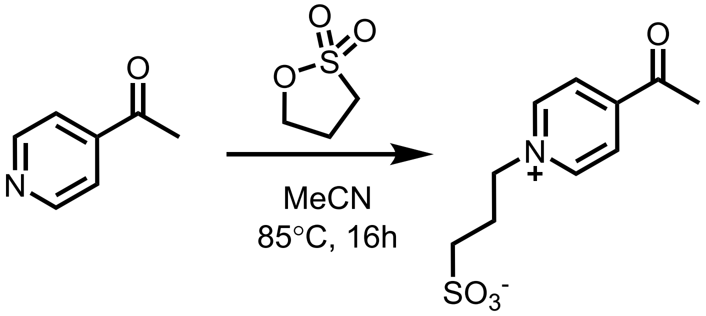

4-Acetylpyridine (2.21 mL, 20 mmol) was dissolved in acetonitrile (15 mL) in a round-bottom flask. While stirring, 1,3-propanesultone (1.76 mL, 20 mmol) was added to the flask. The flask was fitted with a condenser, placed in an oil bath pre-heated to 85 ^{o}C, and left to stir at reflux. After 16 hours, the precipitate was collected by vacuum filtration, washed with acetonitrile, and dried under vacuum to afford AcSP as a light yellow powder (4.84 g, 99%).

^{1}H NMR (400 MHz, D2O) δ 9.20 (d, J = 6.9 Hz, 2H), 8.56 (d, J = 6.9 Hz, 2H), 4.94 (t, J = 7.5 Hz, 2H), 3.06 (t, J = 7.3 Hz, 2H), 2.86 (s, 3H), 2.57 (p, J = 7.3 Hz, 2H). ^{13}C NMR (101 MHz, D_2O) δ 197.85, 149.03, 146.19, 126.60, 60.35, 47.11, 26.76, 26.26. MS (ESI) m/z: calcd for $[C_{10}H_{14}NO_4S]^+$: 244.06; found: 244.06.

Table S1. Summary of FTIR bands of pyridinium redox mediators

Assignment	Band center (cm^{-1})		
	**AcSP**	**BzM**	**BzSP**
ν(N-H)	3115	3093	3106
ν(C-H)	3047	3014	3049
ν(CH_2)	2800-3000		2800-3000
ν(C=O)	1700	1662	1657
ν(C=N)	1640	1596	1596
ν(C=C)	1571	1566	1566
δ(C-H)	1461	1440	1454
ν(S-O)	1180		1180

Table S2. Comparison of performance and energy efficiency of different redox mediators for pH-swing CO_2 capture.

Mediator	Redox center	Cell potential / V		Energy efficiency / kJ/molCO_2	Reference
		**reduction**	**oxidation**		
**BzSP**	pyridinium	0.8 ~ 1.0	1.4 ~ 1.6	102 for reduction 136 for oxidation	This work
**DSPZ**	phenazine	1.2 ~ 1.5	0.5 ~ 0.8	50 for whole cycle	[2]
**1,8-ESP**	phenazine	0.8 ~ 1.2	0.4 ~ 0.8	52 for whole cycle	[3]
**1,5-BTMAPAQ**	quinone	0.9 ~ 1.0	0.1 ~ 0.3	48 ~ 140 for whole cycle	[4]
**1-AP nitrate**	pyridinium	N/A	N/A	101 for whole cycle	[5]

Supporting Figures

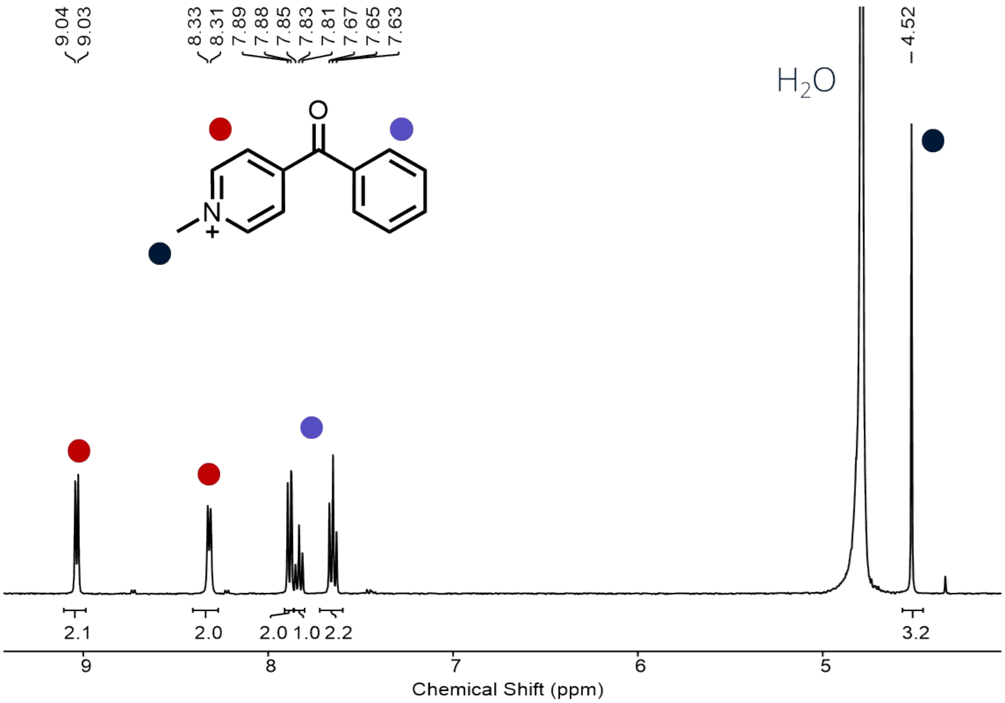

Figure S1. ^{1}H NMR (400 MHz, D_2O) spectrum of BzM.

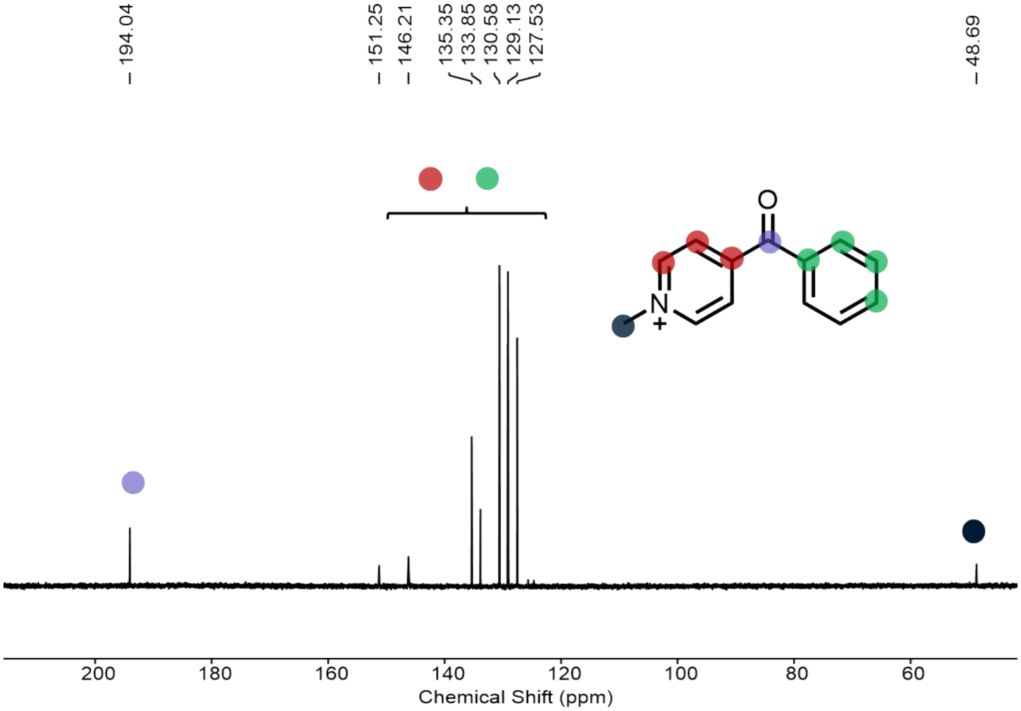

Figure S2. ^{13}C NMR (100 MHz, D_2O) spectrum of BzM.

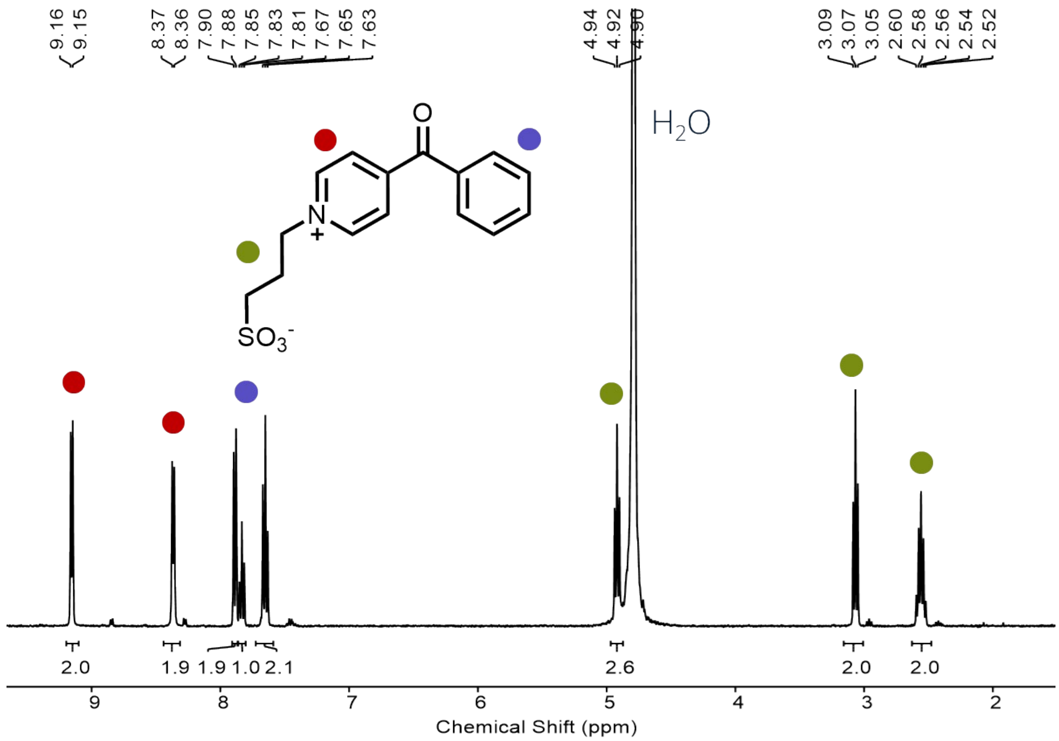

Figure S3. ^{1}H NMR (400 MHz, D_2O) spectrum of BzSP.

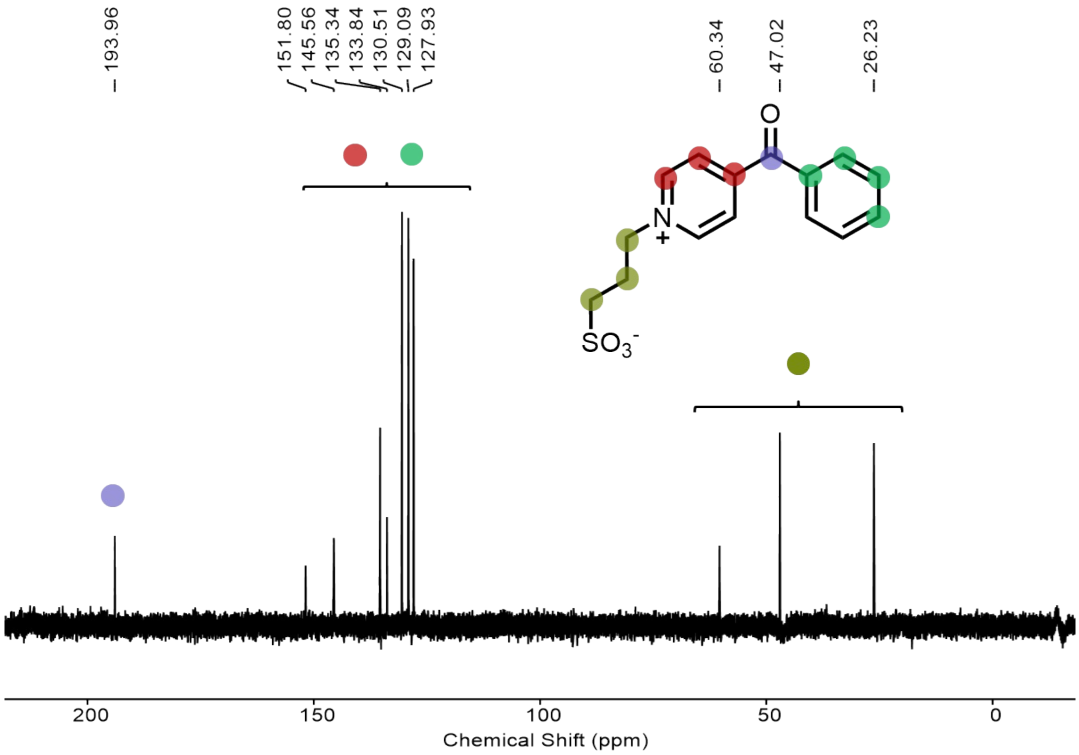

Figure S4. ^{13}C NMR (100 MHz, D_2O) spectrum of BzSP.

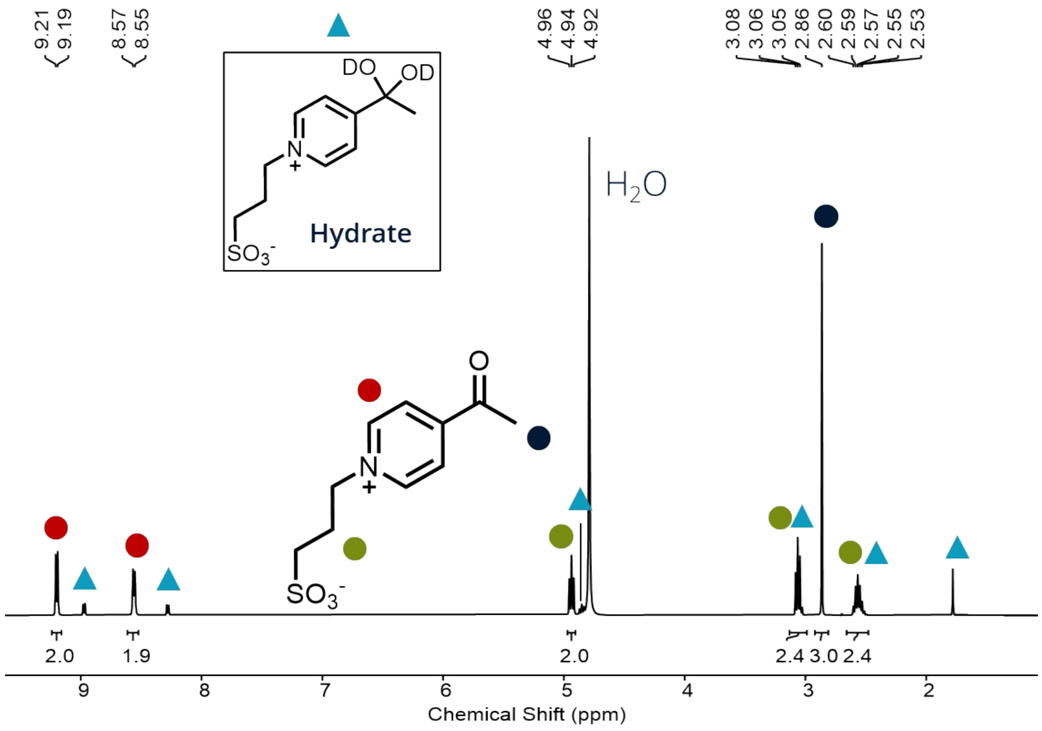

Figure S5. ^{1}H NMR (400 MHz, D_2O) spectrum of AcSP. The additional set of peaks is attributed to the ketone hydrate.[1]

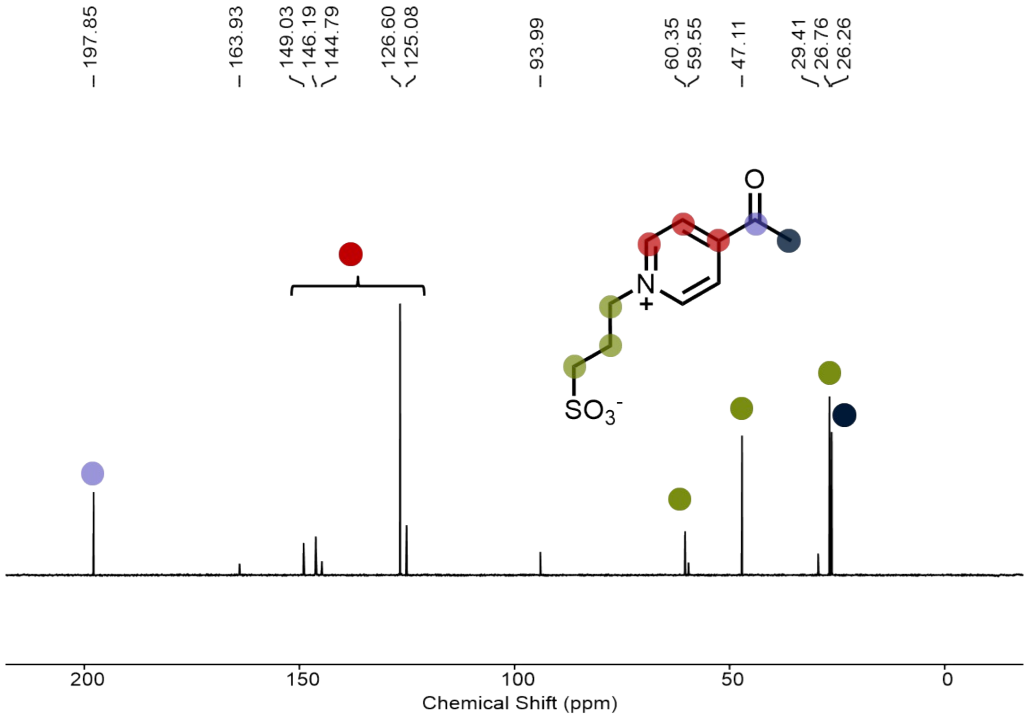

Figure S6. ^{13}C NMR (100 MHz, D_2O) spectrum of AcSP. The additional set of peaks is attributed to the ketone hydrate.[1]

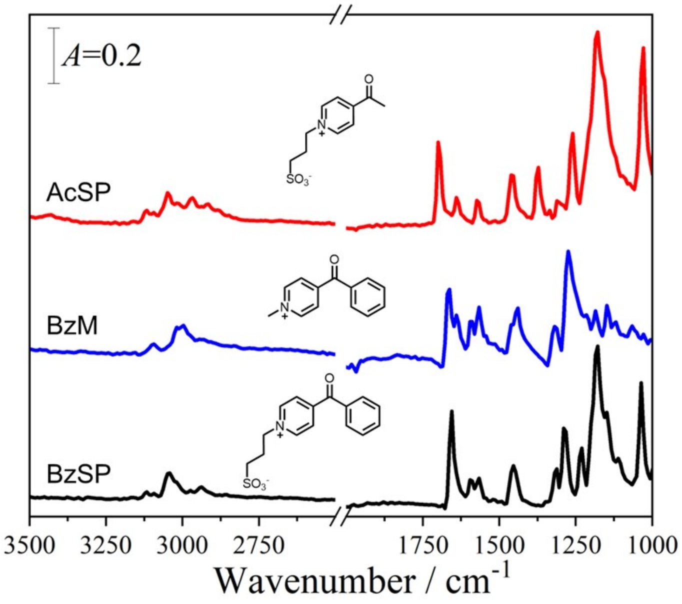

Figure S7. FTIR spectra of AcSP, BzM, and BzSP.

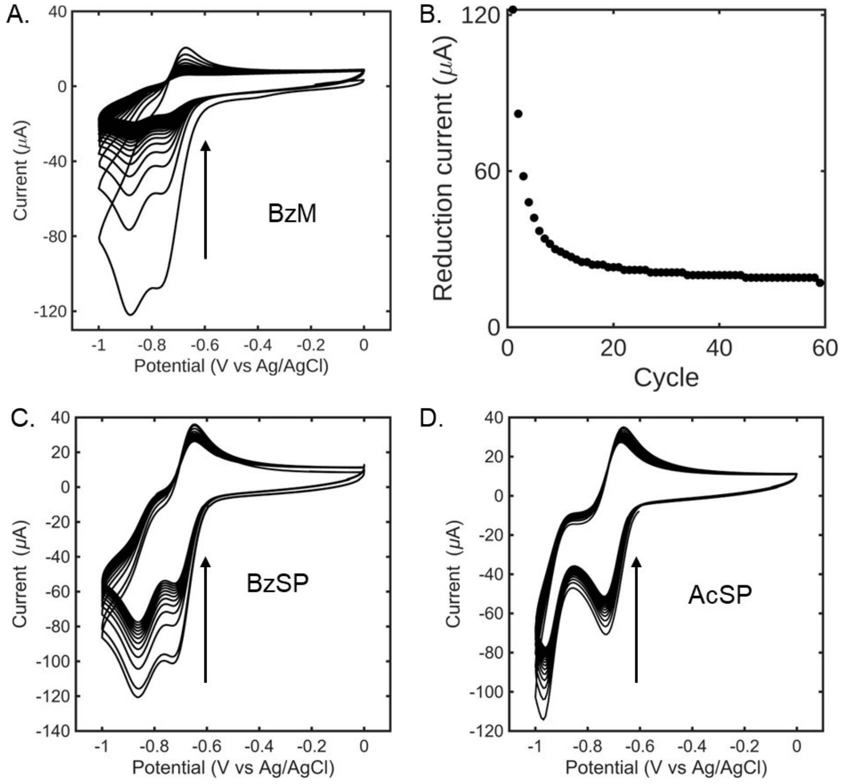

Figure S8. Repeated voltammograms of BzM in a wide window to -1.0 V vs Ag/AgCl, showing poor reversibility when two cathodic events occur (A). Decay in peak cathodic current over 60 cycles (B). Voltammograms of BzSP (C) and AcSP (D) with a wide potential window, also showing two cathodic events and poor reversibility. Scan rate is 0.1 V/s for all voltammograms.

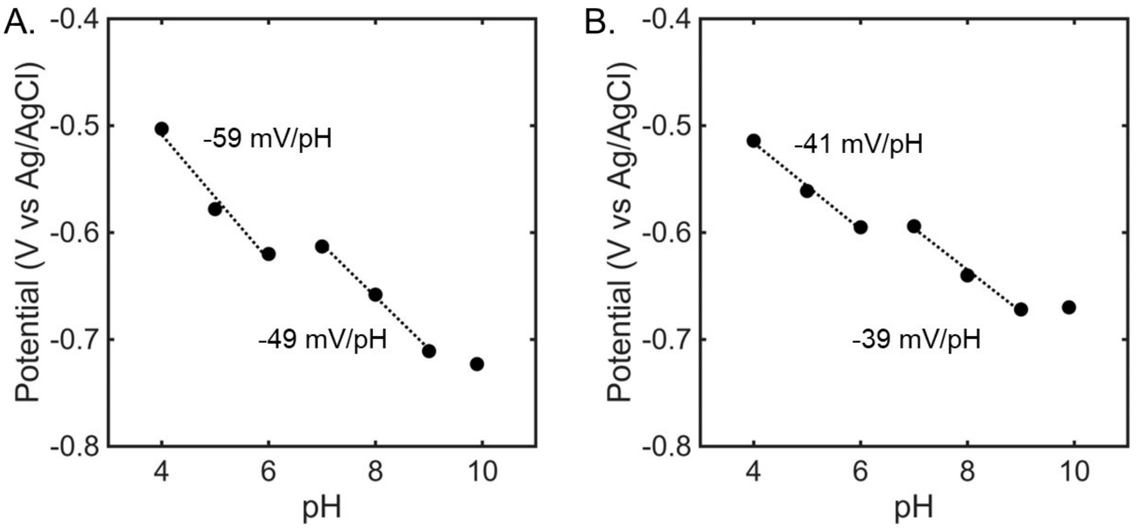

Figure S9. Dependence of reduction potential on pH for BzSP (A) and AcSP (B).

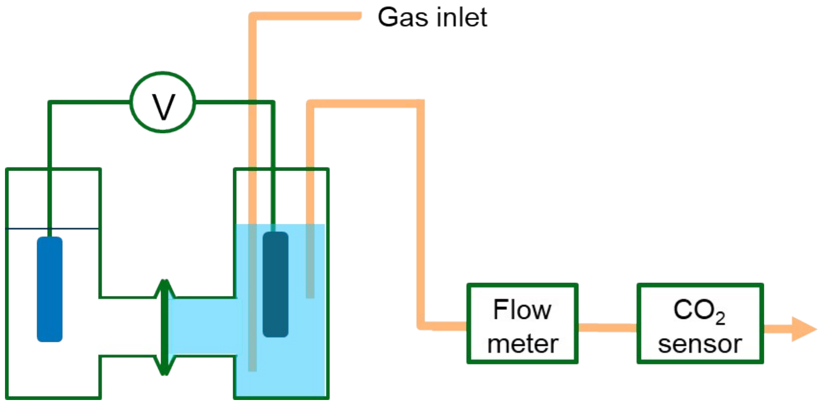

Figure S10. H-cell system used for initial CO_2 capture/release tests.

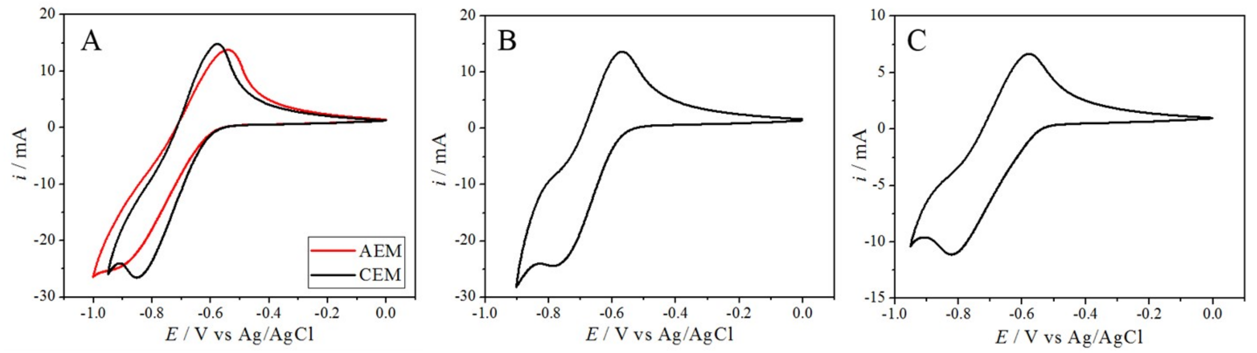

Figure S11. (A) CVs of 0.1 M BzM in 1 M KNO_3 measured in the H-cell using Fumasep AEM (red) and Nafion CEM (black). (B & C) CVs of 0.1 M BzSP (B) and AcSP (C) in 1 M KNO_3 measured in the H-cell.

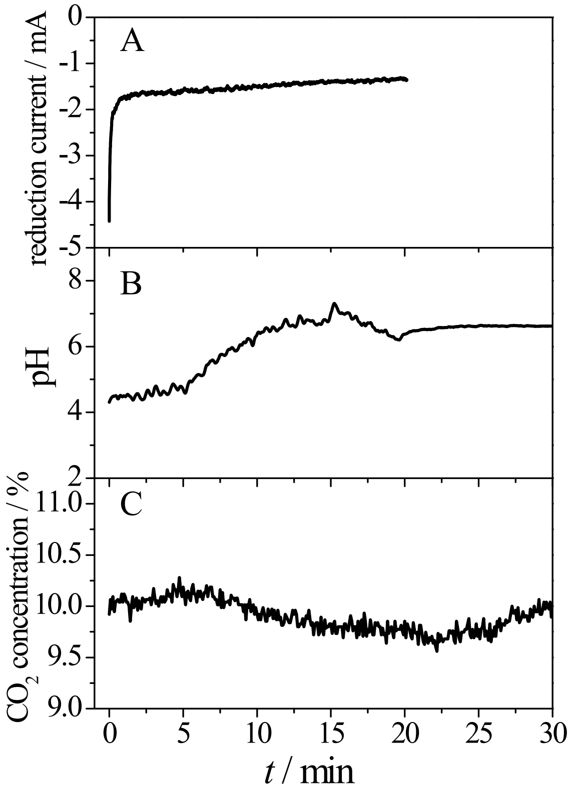

Figure S12. CO_2 capture test using 1 M KNO_3 in the WE compartment of the H-cell. (A): The reduction current at the potential of -0.85 V. (B & C): Variation of the pH of electrolyte in the WE compartment (B) and CO_2 concentration in the gas outlet (C) as a function of time.

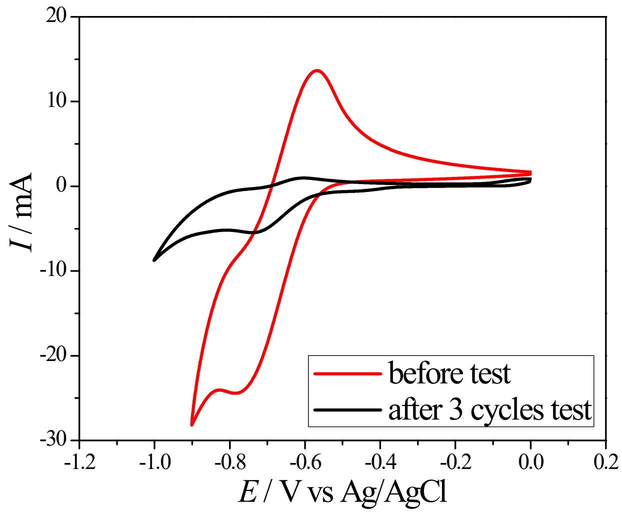

Figure S13. CVs of 0.1 M BzSP in 1 M KNO_3 before (red) and after 3 cycles of CO_2 capture/release test (black) measured in the H-cell.

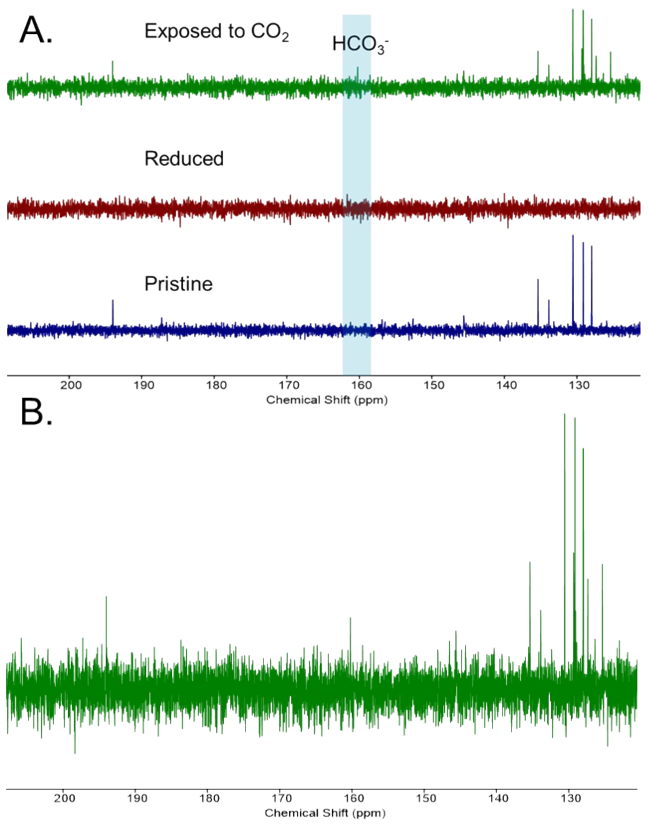

Figure S14. (A) ^{13}C NMR spectrum (151 MHz, D_2O) of BzSP electrolyte in pristine state (blue), following reduction of BzSP to the radical (red), and following exposure of the solution to CO_2 (green). The capture of CO_2 as HCO_3^- is supported by a new peak at 160.2 ppm. (B) Magnified spectrum of CO_2-exposed solution.

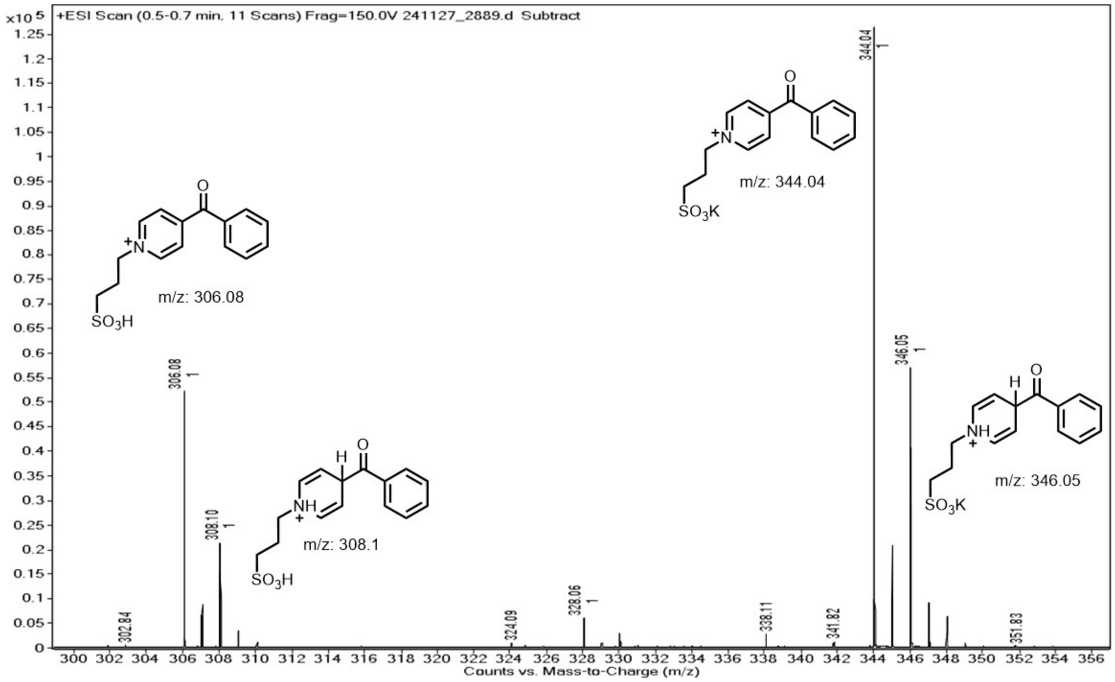

Figure S15. Mass spectrum of cycled BzSP electrolyte, showing the presence of dihydropyridine degradation product.

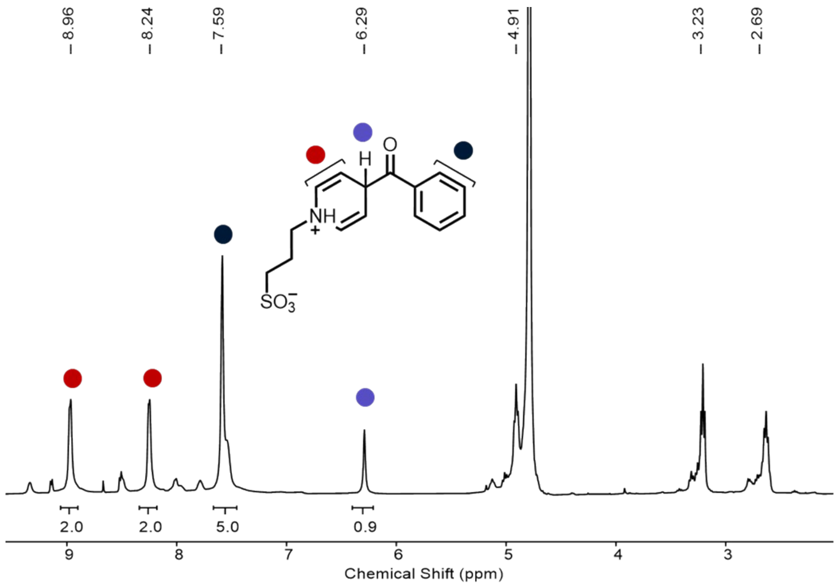

Figure S16. ^{1}H NMR spectrum (D_2O, 400 MHz) of recovered product from spent BzSP electrolyte (0.1 M BzSP in 1 M KNO_3, H_2O as the solvent to avoid deuterium exchange). The peak at 6.29 ppm is indicative of dihydropyridine formation.

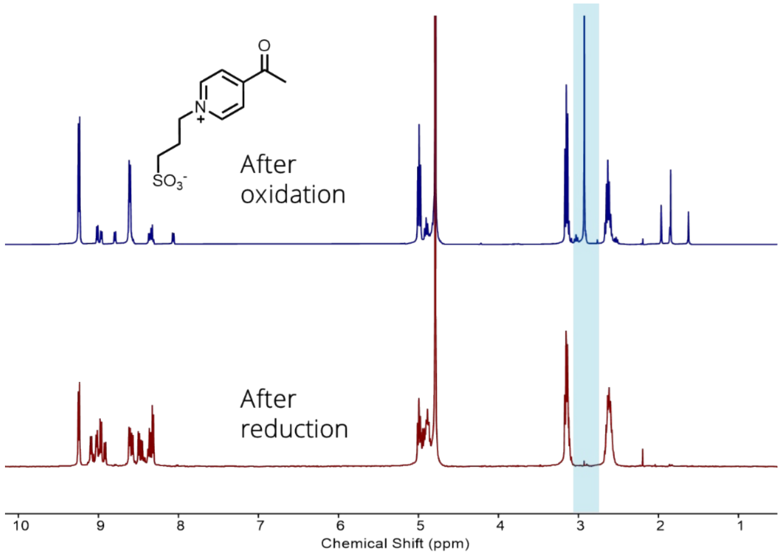

Figure S17. ^{1}H NMR spectrum (D_2O, 400 MHz) of AcSP electrolyte during cycling in D_2O. The acetyl signal disappears in the reduced species, suggesting enolization and subsequent deuterium exchange with the solvent.

References

(1) Huang, S.; Miller, A. K.; Wu, W. Substantial Formation of Hydrates and Hemiacetals from Pyridinium Ketones. *Tetrahedron Letters* **2009**, *50* (47), 6584–6585. https://doi.org/10.1016/j.tetlet.2009.09.054.

(2) Jin, S.; Wu, M.; G. Gordon, R.; J. Aziz, M.; G. Kwabi, D. pH Swing Cycle for CO 2 Capture Electrochemically Driven through Proton-Coupled Electron Transfer. *Energy & Environmental Science* **2020**, *13* (10), 3706–3722. https://doi.org/10.1039/D0EE01834A.

(3) Pang, S.; Jin, S.; Yang, F.; Alberts, M.; Li, L.; Xi, D.; Gordon, R. G.; Wang, P.; Aziz, M. J.; Ji, Y. A Phenazine-Based High-Capacity and High-Stability Electrochemical CO2 Capture Cell with Coupled Electricity Storage. *Nat Energy* **2023**, *8* (10), 1126–1136. https://doi.org/10.1038/s41560-023-01347-z.

(4) Jing, Y.; Amini, K.; Xi, D.; Jin, S.; Alfaraidi, A. M.; Kerr, E. F.; Gordon, R. G.; Aziz, M. J. Electrochemically Induced CO2 Capture Enabled by Aqueous Quinone Flow Chemistry. *ACS Energy Lett.* **2024**, *9* (7), 3526–3535. https://doi.org/10.1021/acsenergylett.4c01235.

(5) Seo, H.; Rahimi, M.; Hatton, T. A. Electrochemical Carbon Dioxide Capture and Release with a Redox-Active Amine. *J. Am. Chem. Soc.* **2022**, *144* (5), 2164–2170. https://doi.org/10.1021/jacs.1c10656.
